# Supplementary material for: Is babesiosis a rare zoonosis in Spain? Its impact on the Spanish Health System over 23 years
Source: PLoS One. 2023 Feb 2;18(2):e0280154. doi: 10.1371/journal.pone.0280154 (PMC9894430; doi:10.1371/journal.pone.0280154)
Supplement: Attachment — Submitted filename: Letter to the authors 08_2022.doc [file pone.0280154.s002.doc]

In the Original Research Article: "IS BABESIOSIS A RARE ZOONOSIS IN SPAIN? ITS IMPACT ON THE SPANISH HEALTH SYSTEM OVER 23 YEARS." Almeida et al. analyzed data provided by hospital discharge records for patients with the diagnosis human babesiosis from 1997 to 2019 in Spain.

The authors found 29 patients diagnosed with babesiosis, 2 of them died. Data provided in the article contain information to kind of diagnosis (principal, primary, secondary), hospital stay, costs, co-morbidities, gender and age.

The study showed an increasing incidence rate over the years, most cases occurred in middle-aged men from urban areas between summer and autumn.

The presenting data are convincing to me for publication as such kinds of studies are rare, if even not existent in

Europe. Nonetheless, I recommend major revisions.

**Comments in detail:**

Abstract:

Background:

The authors write that 2.35% of hospitalized patients in Spain had the diagnosis babesiosis. I cannot imagine that this is right, because it would mean that if 29 patients are 2,35%, altogether only 1234 patients had been hospitalized in Spain (100%)? Please explain what does it mean: ”The incidence of babesiosis in hospitalized patients in Spain is 2.35%.”

Main findings and conclusions:

The content is repeating, e.g. mortality rate, primary diagnosis,.. Please focus and shorten the abstract. Last sentence: “ 100% of fatal cases with secondary diagnosis” – I think you should not write 100% because the number of patients who died is only 2. Better: “Both patients who died,…”

Introduction

The introduction is too long. Please shortened it, e.g. details on ticks, clinic of the disease,.. There are god reviews you can refer to, e.g.

The therapy is not only atovaquone + azithromycin, but also clindamycin + quinine, dependant on the species and severity of the disease.

The authors write that since 2003 only 4 diagnosed cases have been published in Spain: 3x *B. divergens*, 1x *B. microti*. Accorging to the review from Hildebrandt et al., 2021 there were at least 5 cases of *B. microti* and 3 cases of *B. divergens* published from Spain since 2003. Please add these references.

Material and methods

Data collection

The passage about the ICD-9 and ICD-10 is too detailed. Please shorten this passage.

Results

Clinical data

The authors write that 23 of the 29 cases were hospital readmissions. What does this mean? Why were these patients readmitted – because of babesiosis or because of another disease? What were the diagnoses these patients were admitted before and what time (days, weeks) counted as readmission?

Discussion

What are the reason that the cited previous study (32) found a lower incidence of babesiosis in Spain, especially with the information that between 2017 and 2019 only 3 additional cases were reported?

There are more than 1 imported cases, see comments above.

How many of the 23 urban cases received blood transfusion, because you write that accessibility of blood transfusion could be a reason for higher incidence in urban areas?

23 patients were readmissions: see comment above: readmissions because of babesiosis or because of another disease? What were the diagnoses these patients were admitted before and what time (days, weeks) counted as readmission? – does it only mean that these patients with readmissions had co-morbidities, e.g. because of age,..

The reported mortality varies according to the species. What species were identified in the 29 patients? – How many patients had species identifications. What were the methods for diagnosis and what therapy got the two patients who died?

**Arsuaga, M., L. M. Gonzalez, C. A. Lobo, F. de la Calle, J. M. Bautista, I. G. Azcarate, S. Puente, and E. Montero. 2016.** First Report of Babesia microti-Caused Babesiosis in Spain. Vector Borne Zoonotic Dis 16: 677-679.

**Arsuaga, M., L. M. Gonzalez, E. S. Padial, A. W. Dinkessa, E. Sevilla, E. Trigo, S. Puente, J. Gray, and E. Montero. 2018.** Misdiagnosis of Babesiosis as Malaria, Equatorial Guinea, 2014. Emerg Infect Dis 24: 1588-1589.

**Asensi, V., L. M. Gonzalez, J. Fernandez-Suarez, E. Sevilla, R. A. Navascues, M. L. Suarez, M. E. Lauret, A. Bernardo, J. A. Carton, and E. Montero. 2018.** A fatal case of Babesia divergens infection in Northwestern Spain. Ticks Tick Borne Dis 9: 730-734.

**de Ramon, C., J. Cid, S. Rodriguez-Tajes, M. J. Alvarez-Martinez, M. E. Valls, J. Fernandez, and M. Lozano. 2016.** Severe Babesia microti infection in an American immunocompetent patient diagnosed in Spain. Transfus Apher Sci 55: 243-244.

**Gonzalez, L. M., S. Rojo, F. Gonzalez-Camacho, D. Luque, C. A. Lobo, and E. Montero. 2014.** Severe babesiosis in immunocompetent man, Spain, 2011. Emerg Infect Dis 20: 724-726.

**Gonzalez, L. M., E. Castro, C. A. Lobo, A. Richart, R. Ramiro, F. Gonzalez-Camacho, D. Luque, A. C. Velasco, and E. Montero. 2015.** First report of Babesia divergens infection in an HIV patient. Int J Infect Dis 33: 202-204.

**Guirao-Arrabal, E., L. M. Gonzalez, J. L. Garcia-Fogeda, C. Miralles-Adell, G. Sanchez-Moreno, N. Chueca, F. Anguita-Santos, L. Munoz-Medina, D. Vinuesa-Garcia, J. Hernandez-Quero, and E. Montero. 2020.** Imported babesiosis caused by Babesia microti-A case report. Ticks Tick Borne Dis 11: 101435.

**Hildebrandt, A., A. Zintl, E. Montero, K. P. Hunfeld, and J. Gray. 2021.** Human Babesiosis in Europe. Pathogens 10.

**Merino, A. 2016.** Blood film findings in severe babesiosis. Br J Haematol 172: 839.

**Smith, R. P., K. P. Hunfeld, and P. J. Krause. 2020.** Management strategies for human babesiosis. Expert Rev Anti Infect Ther 18: 625-636.
