# Supplementary material for: Is babesiosis a rare zoonosis in Spain? Its impact on the Spanish Health System over 23 years
Source: PLoS One. 2023 Feb 2;18(2):e0280154. doi: 10.1371/journal.pone.0280154 (PMC9894430; doi:10.1371/journal.pone.0280154)
Supplement: Attachment — Submitted filename: Response to ReviewersV2.doc [file pone.0280154.s003.doc]

**Response to Editor**

1. I would suggest deleting figure 2 and briefly describing the data (infection prevalence in relation to the month) in the text.

Thank you very much for your suggestion. We have deleted figure 2 and included a brief description in the text.

**Response to Reviewer**

The manuscript "PONE-D-22-19205R1" analyses the cases of human Babesiosis reported in Spain from 1997 to 2019. Authors analyzed 29 cases, with two deaths, and also reported that most cases were from men patients. In my opinion, the data is worth to be published, however, there are some points in the current version that needs to be revised by authors before it becomes suitable for publication in PLOS ONE.

Abstract

2. In the conclusions section of the abstract authors wrote "Given the low rate of primary diagnoses (55.2%) and the high number of readmissions (79.3%), a low clinical suspicion is likely. There was a 6.9% mortality in our study. Both patients who died were patients with secondary diagnoses of the disease." In my view this should be maintained in the main findings section as results should not appear for the first time in the conclusions.

Thank you very much for your suggestion. We have changed it: we also included the data in the main fingings section.

Introduction

3. In the first and second lines of the introduction I suggest to change "...and transmitted by" by ", which is mainly transmitted by"

Thank you very much for your suggestion. We have changed it.

4. I suggest to delete the sentence "Ticks are parasites found worldwide that have great epidemiological and clinical importance; especially in terms of human health (3,4)and veterinary medicine, affecting 80% of the world´s cattle population (5)." as it does not add any relevant information, and seems to be a lost sentence as ticks are not the main topic of the manuscript.

Thank you, we have deleted this sentence.

5. In the last line of the first paragraph of introduction replace "Ixodes species" by "tick species"

Thank you very much for your suggestion. We have changed it.

6. In first lines of the second paragraph of introduction, please specify in which hosts these prevalence were recorded.

Thank you very much for your suggestion. We have included it. The main hosts were cattle roe deer and small mammals, like mice.

Discussion

7. In the third paragraph of discussion, please add "sensu lato" after "Borrelia burgdorferi".

Thank you very much for your suggestion. We have included it.

8. Still in the third paragraph of discusion the authors wrote "Of the 29 patients included in our study, only 16 of them (55.2%) had a primary diagnosis of human babesiosis on their discharge report." I'm not sure that it is a low number, more than half of the patients had babesiosis as primary diagnosis. Does the authors checked if this number should be considered low. Is there data for other diseases that may explain why it should be considered low? In addition, the sentence "leads us to think of a very low clinical suspicion is likely human babesiosis" is not clear and shoud be rephrased.

Thank you very much for your suggestion. We did not check whether this number should be considered low. We made the changes in the text.

9. Please delete "We have also to declare that" from the fifth paragraph and start the sentence with "The death of these patients..."

Thank you, we have corrected this mistake in the text.

Yours sincerely, Moncef Belhassen Garcia
